# MiR-133a is downregulated in non-small cell lung cancer: a study of clinical significance

**DOI:** 10.1186/s40001-015-0139-z

**Published:** 2015-04-23

**Authors:** Dong Lan, Xin Zhang, Rongquan He, Ruixue Tang, Ping Li, Qiancheng He, Gang Chen

**Affiliations:** Department of Medical Oncology, First Affiliated Hospital of Guangxi Medical University, No. 6 Shuangyong Road, Nanning, Guangxi Zhuang Autonomous Region 530021 People’s Republic of China; Department of Pathology, First Affiliated Hospital of Guangxi Medical University, No. 6 Shuangyong Road, Nanning, Guangxi Zhuang Autonomous Region 530021 People’s Republic of China; Department of General Medicine, First Affiliated Hospital of Wenzhou Medical University, No. 2 Fuxue Alley, Wenzhou, Zhejiang 325000 People’s Republic of China

**Keywords:** MiR-133a, NSCLC, Downregulate, Clinical significance

## Abstract

**Background:**

Despite present studies which suggested miR-133a as a promising biomarker for several cancers, there still exist no articles concerning the validated clinical significance of miR-133a in non-small cell lung cancer (NSCLC). Therefore, in this study, we targeted the correlation between miR-133a expression and clinicopathological significance in NSCLC patients.

**Methods:**

The expression of miR-133a in 125 cases of NSCLC and their paired adjacent non-cancerous tissues was evaluated by quantitative reverse transcription polymerase chain reaction (qRT-PCR). Meanwhile, the relationship between miR-133a expression and several clinicopathological parameters and patient survival was analyzed.

**Results:**

The relative level of miR-133a was 2.0108 ± 1.3334 in NSCLC tissues, significantly lower than that of the adjacent non-cancerous lung tissues (3.6430 ± 2.2625, *P* = 0.019). The area under curve (AUC) of low expression of miR-133a to diagnose NSCLC was 0.760 (95% CI: 0.702 ~ 0.819, *P* < 0.001). MiR-133a expression was negatively correlated to lymphatic metastasis (*r* = −0.182, *P* = 0.042), tumor size (*r* = −0.253, *P* = 0.04), clinical TNM stages (*r* = −0.154, *P* = 0.087), and EGFR protein expression (*r* = −0.612, *P* < 0.001).

**Conclusions:**

MiR-133a serves as a tumor-suppressive miRNA in human NSCLC, and its downregulation suggests deterioration in NSCLC patients.

## Background

Lung cancer is the major cause of cancer mortality worldwide with an approximation of 80% non-small cell lung cancer (NSCLC) [1-3]. Although tremendous improvements have been made in diagnosis and treatment, poor prognosis still exists in a large number of NSCLC patients with a low 5-year overall survival rate and a high recurrence rate [4-6]. Thus, it is utterly important to discover reliable biomarkers of profound prognostic value in NSCLC patients [7].

MicroRNAs (miRNAs) are short single-stranded non-coding RNA molecules containing about 19 to 25 nucleotides, which regulate RNA silencing and post-transcriptional regulation of gene expression [8,9]. The malfunction of miRNAs is frequent in different carcinomas and plays a significant role in cancer progression [10-12]. It is generally believed that certain miRNAs, which are aberrantly expressed, can serve as potential biomarkers in terms of diagnosis and prognosis [13-16]. Recent studies about miRNAs in NSCLC unveiled that certain deregulated miRNAs are associated with the regulation of cell growth, apoptosis, migration, and invasion in NSCLC, such as miR-34a, miR-125a, miR-145, miR-451, and miR-17-92 cluster [17,18]. The discoveries suggested that the dysregulation of miRNA expression might be strongly associated with oncogenesis and progression of NSCLC.

The expression of miR-133a was reported to be downregulated in various malignancies when cancerous tissue was compared with normal adjacent tissue, including bladder cancer, head and neck squamous cell carcinoma, and colorectal cancer [19-21]. Also, the aberrant expression of miR-133a emerged among breast cancer, renal cell carcinoma, and prostate cancer [22-24]. Nevertheless, to date, there are very few studies attempting to expound the relationship between the expression of miR-133a and the clinicopathological parameters in NSCLC, except the research by Wang et al. [25], in which only Kaplan-Meier survival rate was taken into account. Further investigations are hence required to define the clinical significance of miR-133a in NSCLC.

## Methods

### Tissue samples

In the current study, we collected the formalin-fixed, paraffin-embedded (FFPE) samples of cancerous and non-cancerous adjacent lung tissues from 125 NSCLC patients (75 males and 50 females; mean age, 61.10 years; range, 23 to 90 years), who were hospitalized in the First Affiliated Hospital of the Guangxi Medical University (Nanning, Guangxi, China) between January 2012 and February 2014. The research was approved by The Ethical Committee of the First Affiliated Hospital of Guangxi Medical University, China, and informed written consent was obtained from all patients who participated. All tissue samples were reviewed and diagnosed by two pathologists independently. The clinicopathological characteristics were summarized in Table 1. The defining criteria for age and pathological grading were consulted from the report by Li et al. [26] Also, the tumor size and TNM definition criterion complies with the gauge from IASLC 2009 [27].

**Table 1:** **Relationship between miR-133a and clinicopathological parameters in NSCLC** $\left(\overline{\mathbf{x}}\pm \mathbf{s}\right)$

**Clinicopathological feature**		***n***	**miRNA-133a relevant expression (2** ^−**Δcq**^ **)**		
			**Mean ± SD**	***t***	***P*** **value**
Tissue^a^	NSCLC	125	2.0108 ± 1.3334	6.949	<0.001
	Adjacent non-cancerous lung	125	3.6430 ± 2.2625		
Age (years)	<60	57	1.8321 ± 1.2583	1.377	0.171
	≥60	68	2.1606 ± 1.3846		
Gender	Male	75	1.9552 ± 1.2893	0.569	0.570
	Female	50	2.0942 ± 1.4060		
Smoke	No	38	1.7792 ± 1.2891	1.565	0.122
	Yes	30	2.3217 ± 1.5689		
Tumor size (cm)	≤3 (pT1)	60	2.3058 ± 1.2512	−2.423	0.017
	>3 (pT2, pT3, pT4)	65	1.7385 ± 1.3581		
Lymph node metastasis	No	56	2.2662 ± 1.3316	−1.951	0.053
	Yes	69	1.8035 ± 1.3079		
Vascular invasion	No	90	2.0787 ± 1.3777	−0.912	0.364
	Yes	35	1.8363 ± 1.2134		
TNM	I-II	54	2.2480 ± 1.3434	−1.749	0.083
	III-IV	71	1.8304 ± 1.3063		
Pathological grading^b^	I	17	2.2965 ± 1.5570	−1.074	0.345
	II	78	1.8772 ± 1.3135		
	III	30	2.1963 ± 1.2459		
Histological classification^c^	Adenocarcinoma	101	2.0638 ± 1.3517	4.980	0.008
	Squamous carcinoma	23	1.6222 ± 0.9795		
	Large cell carcinoma	1	5.6000 ± 0.0000		
EGFR amplification	No	39	2.0159 ± 1.3763	−0.889	0.378
	Yes	18	1.6533 ± 1.5483		
EGFR protein expression	Low	40	2.2843 ± 1.3288	−3.379	0.001
	High	17	1.0006 ± 1.2706		
EGFR mutation	Wild type	44	1.9625 ± 1.4570	−0.590	0.557
	Mutation^d^	13	1.6946 ± 1.3646		

^a^Paired *t* student’s test was performed.

^b^One-way analysis of variance (ANOVA) test was performed.

^c^Respective results of comparisons in histological classification were described as below:

Adenocarcinoma *vs* squamous carcinoma (*t* = 1.479, *P* = 0.142).

Adenocarcinoma *vs* large cell carcinoma (*t* = −2.603, *P* = 0.011).

Squamous carcinoma *vs* large cell carcinoma (*t* = −3.976, *P* = 0.001).

^d^EGFR mutation included short in-frame deletions in exon 19 and point mutations that result in a substitution of arginine for leucine at codon 858 (L858R) in exon 21.

### EGFR status detection

We detected the EGFR status in the way previously reported [28]. Briefly, for immunohistochemistry (IHC) to detect EGFR protein expression, NSCLC tissue sections were de-paraffinized and antigen retrieval was performed with citrate buffer (pH 6.0) with 0.05% Tween-20. Slides were incubated with primary EGFR polyclonal antibody (sc-03, Santa Cruz Biotechnology, Inc., Santa Cruz, CA, USA) at 4°C overnight. The expression level of EGFR protein was documented with the quickscore (Q score) which is based on the percentage (*P*) of positive staining tumor cells (0% to 100%) and the intensity (*I*) of staining (0, complete nonappearance of staining; 1, weak cytoplasmic staining; 2, moderate and incomplete membranous staining; 3, solid membranous staining). Both the intensity and percentage of staining were assessed with objective magnification × 10, while the distribution of staining on membrane or cytoplasm was evaluated with objective magnification × 40. The result of each case was calculated by multiplying the percentage of positive tumor cells with the intensity (*Q* = *P* × *I*; maximum = 3). When Q score was ≥1, the result was regarded as positive. The Q score ≤2 was considered as low expression of EGFR, while >3 was high expression. An overview of all the IHC results was performed by two independent pathologists (GC and PL). Two persons estimated the staining individually, and discrepancies were determined by consensus.

Concerning the EGFR gene amplification, gene copy number per cell in NSCLC was investigated by FISH. The LSI EGFR Spectrum Orange/CEP7 Spectrum Green probe (Vysis, Abbott Laboratories, Chicago, IL, USA) was used following the manufacturer instructions. FISH signals were assessed under the fluorescence microscope Olympus BX41 (Olympus, Tokyo, Japan) equipped with single filters: DAPI, SpectrumOrange, and FITC as well as a triple-filter DAPI/FITC/SpectrumOrange. FISH analysis was individualistically performed by two pathologists who were unaware of the clinicopathological and molecular features of patients. Negative results for FISH in NSCLCs were determined if it was with no or low genomic gain (≤four copies of gene in >40% of the cells), and positive results included gene amplification and high polysomy. Gene amplification was defined by the presence of tight gene clusters, a gene/chromosome per cell ratio ≥2, or ≥15 copies of the genes per cell in ≥10% of the analyzed cells, and high polysomy was identified as ≥ four copies of the gene in ≥40% of the cells.

As for the EGFR mutation detection, the QIAamp DNA FFPE Tissue Kit (QIAGEN, Hilden, Germany) was used to extract DNA from paraffin-embedded tissues and the operational tumor samples with histological control for the presence of tumor cells (>75%) that was obtained by trimming the non-cancerous tissue and necrotic tissue. For mutational analysis of the kinase domain of EGFR coding sequence, exons 18, 19, 20, and 21 were amplified with specific pairs of primers, specific to the flanking sequences of individual exon with the EGFR reference sequence (NM_005228.3, NCBI). The assay was carried out according to the manufacturer’s protocol with the ABI Step-one Plus real-time PCR system.

### RT-qPCR

RNA isolation and RNA normalization were performed as described formerly [29]. We applied reverse transcription (RT) and qPCR kits based on precedents in order to examine the expression of miR-133a as reported previously [30]. RT process for microRNA complied strictly with the instructions of the manufacturer. Previously, we discovered that the aggregation of miR-191 and miR-103 was the most stable housekeeping miRNA by using NormFinder and geNorm. This combination was adopted in the current study for the evaluation of miR-133a expression. The primers for miR-133a, miR-191, and miR-103 were included in TaqMan® MicroRNA Assays (4427975, Applied Biosystems, Life Technologies, Grand Island, NY, USA). Sequences of targeted miRNAs and reference miRNAs used were as follows: miR-133a (Applied Biosystems Cat. No. 4427975-000458): UUGGUCCCCUUCAACCAGCUGU; miR-191 (Applied Biosystems Cat. No. 4427975-000490): CAACGGAAUCCCAAAAGCAGCU; miR-103 (Applied Biosystems Cat. No. 4427975-000439): AGCAGCAUUGUACAGGGCUAUGA. The reverse primers were also used for the reverse transcription with TaqMan® MicroRNA Reverse Transcription Kit (4366596, Applied Biosystems, Life Technologies, Grand Island, NY, USA) in a total volume of 10 μl. We carried out real-time qPCR for miRNA using Applied Biosystems PCR7900. The expression of miR-133a was determined with the formula 2^−Δcq^ [31].

### Statistical analysis

We employed SPSS 20.0 for statistical analysis. Student’s *t* test was conducted to discover the significance of difference between groups. We also adopted one-way analysis of variance (ANOVA) test to identify the relationship between the expression level of miR-133a and pathological grading and histological classification. Receiver operating characteristic (ROC) curves were created by SPSS 20.0 to evaluate how potent miR-133a is when it came to distinguish the NSCLC tissues from non-cancerous lung tissues. Survival analysis was gauged by the Kaplan-Meier method while the log-rank test was conducted to compare the survival status between groups. It was considered to be statistically significant when the *P* value calculated by two-tailed test was less than 0.05.

## Results

### Decreased expression of miR-133a in NSCLC

**Figure 1:**
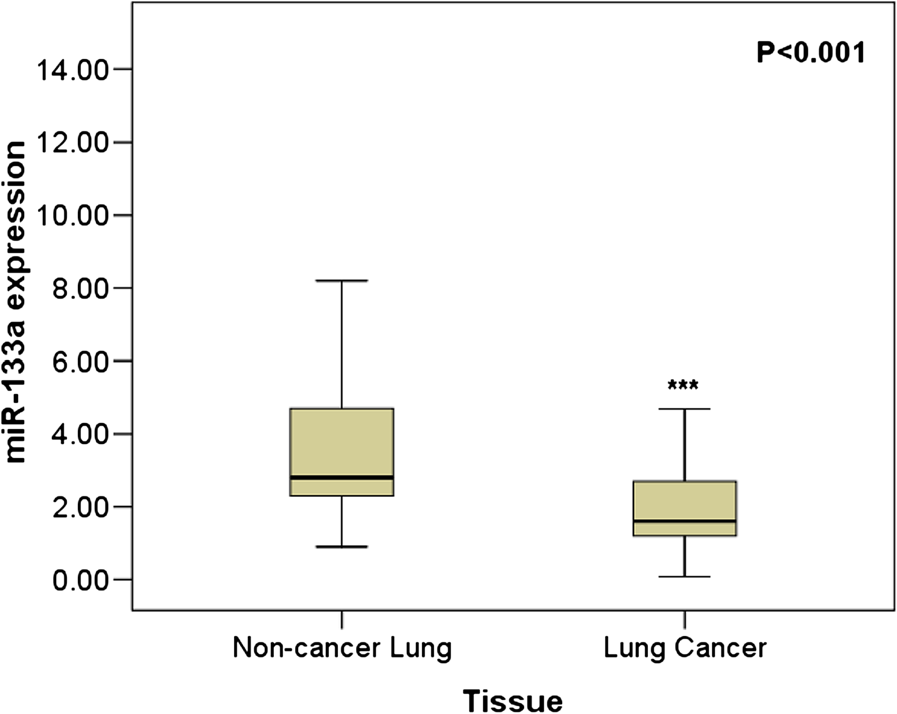
The expression of miR-133a in lung cancer and non-cancerous lung tissues. qRT-PCR was employed to detect the expression of miR-133a in lung cancer tissue and adjacent non-cancerous lung tissue. *** *P* **<** 0.001.

**Figure 2:**
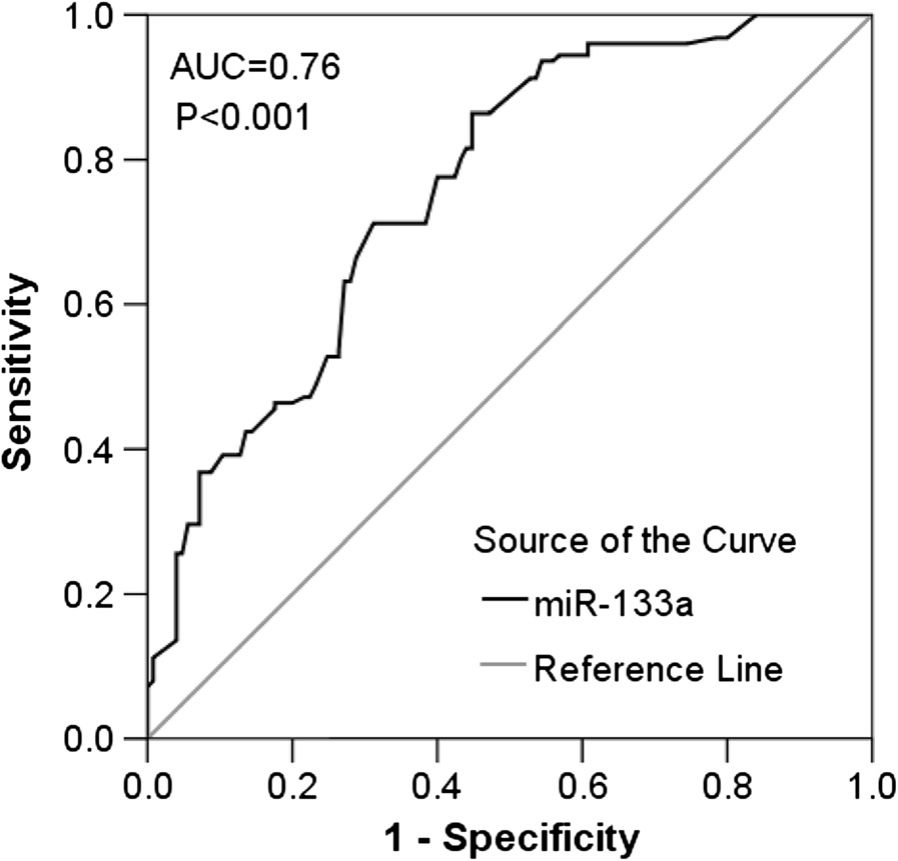
ROC curve of miR-133a for lung cancer. The area under curve (AUC) of miR-133a was 0.760 (95% CI: 0.702 ~ 0.819, *P* < 0.001).

The relative level of miR-133a in NSCLC tissues was 2.0108 ± 1.3334, which was significantly lower than that in the adjacent non-cancerous lung tissues (3.6430 ± 2.2625, *P* = 0.019, Figure 1 and Table 1). Moreover, the ROC curve was applied in order to evaluate the diagnostic performance of miR-133a in NSCLC. The area under curve (AUC) of miR-133a was 0.760 (95% CI: 0.702 ~ 0.819, *P* < 0.001, Figure 2), and the optimal cut-off value was 1.690.

### Correlations between the miR-133a expression and clinicopathological parameters in NSCLC

**Figure 3:**
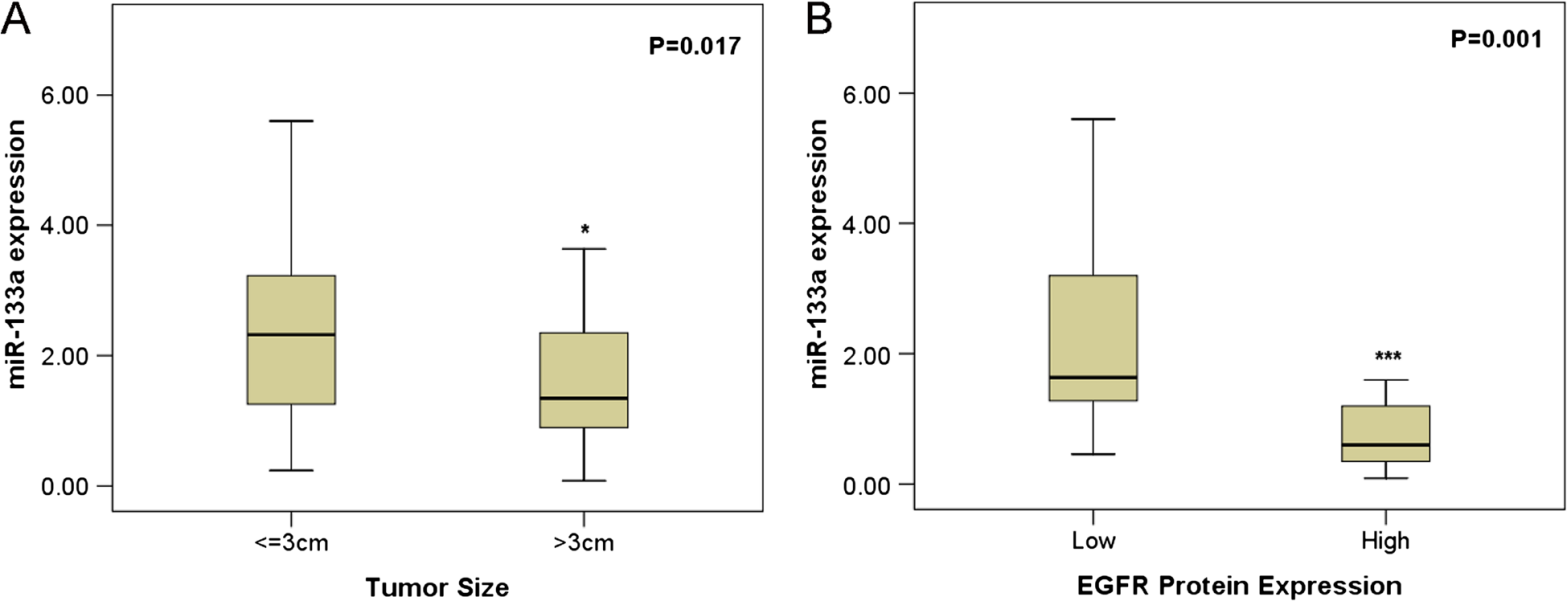
Correlations between the expression of miR-133a and some clinicopathological parameters in lung cancer. **(A)** tumor size; **(B)** EGFR protein expression. *P < 0.05.

MiR-133a was identified to be associated with certain clinicopathological parameters. The relative level of miR-133a expression in patients with tumor greater than 3 cm (1.7385 ± 1.3581) was significantly lower when compared to that in those with tumor less than or equal to 3 cm (2.3058 ± 1.2512, *P* = 0.017, Figure 3A). We also observed certain differences in histological classification, with the expression level 2.0638 ± 1.3517 in adenocarcinoma, 1.6222 ± 0.9795 in squamous carcinoma, and 5.6000 ± 0 in large cell carcinoma. Statistical significance existed in situations where we compared adenocarcinoma with large cell carcinoma (*P* = 0.011) and squamous carcinoma with large cell carcinoma (*P* = 0.001). As for the association between miR-133a level and EGFR status, we first detected EGFR expression by IHC. All 57 cases assessed for IHC showed positive EGFR staining with the Q score more than 1. Among them, 40 had low expression and 17 had high expression of EGFR. Relatively lower level of miR-133a expression was also perceived in cases of high EGFR protein expression (2.2843 ± 1.3288) while higher level was detected in those with low EGFR protein expression (1.0006 ± 1.2706, *P* = 0.001, Figure 3B). Even though considered to be insignificant statistically, in NSCLC patients with lymphatic metastasis, we detected a higher level of miR-133a, that is, 2.2662 ± 1.3316 while there was a lower level in those without lymphatic metastasis, namely 1.8035 ± 1.3079 with the *P* value 0.053. Furthermore, a decreasing trend of miR-133a could be found in the clinical TNM stages. The expression of miR-133a in advanced stages (III and IV, 1.8304 ± 1.3063) was relatively decreased when compared with that in early stages (I and II, 2.2480 ± 1.3434, *P* = 0.083).

Meanwhile, Spearman correlation test was employed for further analysis, which revealed the consistent relationship between miR-133a expression and the following clinicopathological parameters: lymphatic metastasis (*r* = −0.182, *P* = 0.042), tumor size (*r* = −0.253, *P* = 0.04), and EGFR protein expression (*r* = −0.612, *P* < 0.001).

Nevertheless, other clinicopathological features which proved to be independent of miR-133a expression were as follows: age, gender, differentiation grades, pathological types, smoke, vascular infiltration, metastasis, EGFR amplification, or EGFR mutation status.

### ROC curves of clinicopathological parameters

**Figure 4:**
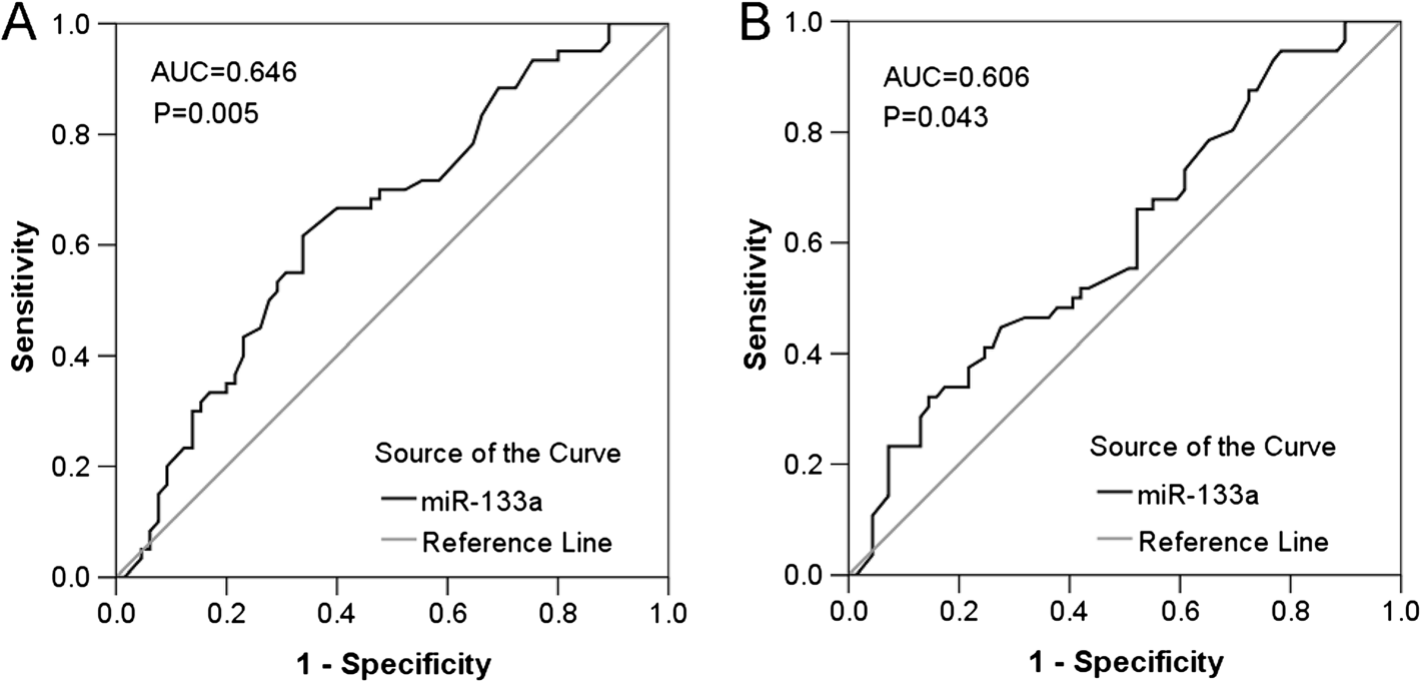
ROC curves of miR-133a for clinicopathological factors of lung cancer. **(A)** ROC curve of miR-133a level for tumor size. The AUC was 0.646 (95% CI: 0.550 ~ 0.743, *P* = 0.005). **(B)** ROC curve of miR-133a level for lymphatic metastasis. The AUC was 0.606 (95% CI: 0.507 ~ 0.705, *P* = 0.043).

ROC curves were applied to evaluate the predictive significance of miR-133a level in NSCLC patients for clinicopathological factors. The AUC in terms of size was 0.646 (95% CI: 0.550 ~ 0.743, *P* = 0.005, cut-off value: 1.620, sensitivity: 61.7%, 1-specificity: 33.8%) (Figure 4A). The area under curve (AUC) in patients with lymphatic metastasis was 0.606 (95% CI: 0.507 ~ 0.705, *P* = 0.043, cut-off value: 2.940, sensitivity: 32.1%, 1-specificity: 14.5%) (Figure 4B). As for other clinicopathological factors, there appeared to be inferior diagnostic significance.

### Overall survival analysis of NSCLC patients

**Figure 5:**
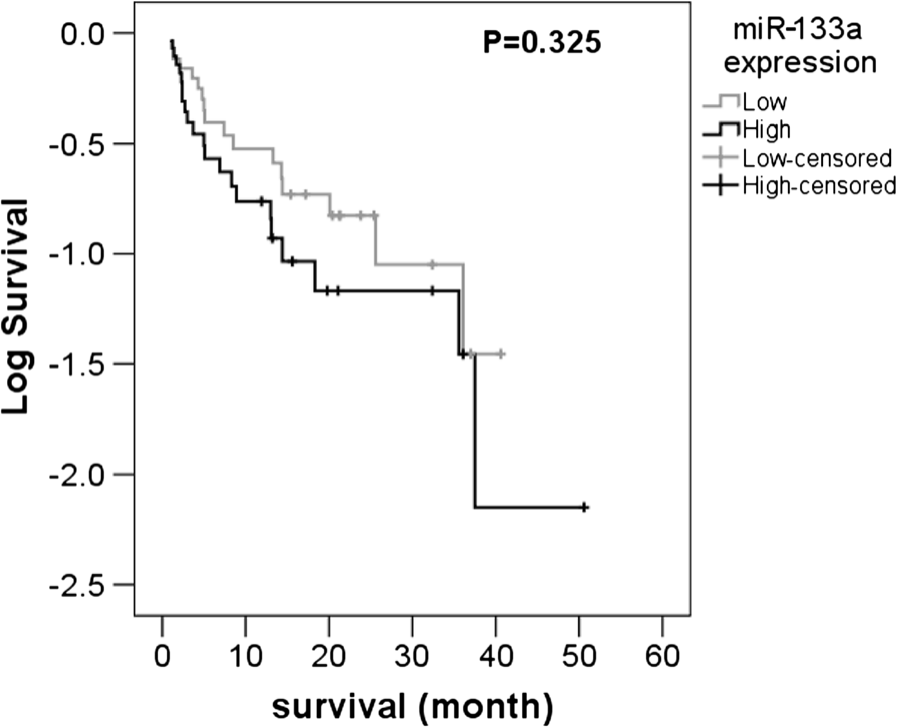
Kaplan-Meier curve for survival in miR-133a expression. No statistical significance of survival emerged in patients with low or high miR-133a expression (*P* = 0.325).

Among the 57 patients followed up, 27 had relatively low miR-133a level (lower than the median level of 1.60) while 30 possessed relatively high level of miR-133a expression. The high miR-133a expression group showed a survival time of 20.012 ± 3.132 months in contrast to 17.296 ± 3.424 months in low miR-133a expression group. It is worth mentioning that there existed a distinct difference of 2.716 months in the survival between the two groups even though no statistical significance of miR-133a expression was shown in survival of NSCLC (*P* = 0.325, Figure 5).

## Discussion

To our knowledge, this study was the first one to illuminate the relationship between miR-133a and clinicopathological parameters in NSCLC. There were only two publications concerning the role of miR-133a in NSCLC, which concentrated more on its regulating mechanism than the clinical significance [25,32]. In the perspective of clinical significance, Wang et al. [25] concluded that miR-133a expression levels indicate the clinical outcome in NSCLC and could serve as a suitable prognostic factor merely based on the multivariable Cox regression analysis. Moriya et al. [32] paid substantial attention to the molecular mechanisms but none to its clinical significance. What makes our current study potent and novel is that we examined the miR-133a expression in relatively larger sample sizes, 125 cases of NSCLC tissues, and their paired non-cancerous lung tissues, which minimized individual difference, and ran a full-panel analysis between the expression levels of miR-133a and clinicopathological parameters in NSCLC.

We found the role of miR-133a as a tumor suppressor in NSCLC. The relative expression level of miR-133a was significantly lower than that in the non-cancerous lung tissues. Moreover, the ROC analysis results demonstrated that miR-133a had a moderate diagnostic value for NSCLC with the AUC of 0.760. Moriya et al. [32] and Wang et al. [25] also reported the suppressive role of miR-133a in NSCLC. In consideration of literatures and the current study [25,32], it strongly suggests the potential tumor-suppressive role of miR-133a and the possibility to be regarded as a promising diagnostic biomarker as well as a target of treatment in NSCLC.

Our main focus in the current study lied in the correlation between miR-133a and clinicopathological parameters in NSCLC. To begin with, miR-133a was significantly downregulated in NSCLC tissues with larger tumor diameter (*P* = 0.017), which unveiled that miR-133a might correlate with the growth of tumor in NSCLC positively. However, although statistically significant, the correlation between tumor size and miR-133a expression was quite weak. Then, we came to the relationship between the miR-133a expression and lymphatic metastasis. A higher level of miR-133a, 2.2662 ± 1.3316, was shown in patients with lymphatic metastasis while a lower level, 1.8035 ± 1.3079, was observed in those without lymphatic metastasis. The difference should never be ignored despite the boardline statistical significance (*P* = 0.053). A larger cohort is needed to further determine the relationships between miR-133a expression and tumor size as well as lymphatic metastasis. It was actually supported by the study of Wang et al. [25], in which they claimed that miR-133a can inhibit cell invasiveness. Furthermore, the expression of miR-133a suggested the deterioration of the disease to certain degree in spite of inferior statistical significance (*P* = 0.083), since the miR-133a expression in advanced stages (III and IV, 1.8304 ± 1.3063) was lower than that in early stages (I and II, 2.2480 ± 1.3434). As to histological classification, we considered it to be fortuitous owing to the sole case of large cell carcinoma, even though there existed a statistical significance in the cases of adenocarcinoma *vs* large cell carcinoma and squamous carcinoma *vs* large cell carcinoma. The above results of the study reveal a remarkable significance between miR-133a and tumor growth, metastasis, and progression of NSCLC. However, further study with a larger size of cohort is required to confirm the current finding.

However, our results seem to contradict Wang et al. [25] with regard to survival. According the their report, the underexpression of miR-133a was significantly associated with poor overall survival with a *P* value of 0.0409, which later inferred that miR-133a could be a prognostic indicator when combined with the results of multivariable Cox regression analyses. However, in our study, there emerged no statistical significance of miR-133a expression in the survival of NSCLC. We assume that four main factors should account for the situation. Firstly, the sample sizes were different with 57 followed-up cases in our study and 112 in theirs. Secondly, the conditions of patients also differed. For instance, all cases of NSCLC patients used to analyze survival in our study were adenocarcinoma, while it was not specified which subtype the 112 cases in the study of Wang et al. [25] were. Thirdly, different calculating methods of relative gene expression might also contribute to the discrepancy. We used the formula 2^−Δcq^ when determining the expression of miR-133a while Wang et al. did not specify the calculating method of gene expression in their article [25]. Last but not least, endogenous controls might result in the difference. In the study of Wang et al. [25], they employed RNU48 as the endogenous control while we adopted a combination of miR-191 and miR-103 as the endogenous control in our research. We hence plan to collect more followed-up cases and their corresponding data for the purpose of further study in terms of survival.

It would be hard for us to neglect the distinct correlation between the miR-133a level and the expression of EGFR protein (*r* = −0.612, *P* < 0.001) as assessed by Spearman’s correlation. They were obviously correlated negatively, which strongly backed the perspective of Wang et al. [25], who assumed that several oncogenic receptors in NSCLC cells might be direct targets of miR-133a, including EGFR. Putative miR-133a binding sites of EGFR have also been identified by computational algorithms from several online miRNA-target gene prediction softwares, including Targetscan (www.targetscan.org, data not shown). Treating strategy using EGFR has gained growing attention, which provides a significant response and survival benefit. Nevertheless, resistance has already emerged regardless of unexpanded usage [33,34]. Thus, it is rational that a trend of resorting to miR-133a as a therapeutic strategy has become increasingly popular. Previously, we found that EGFR was a target gene of miR-146a in NSCLS and we attempted to investigate the effect of miR-146a in the treatment of NSCLC. MiR-146a did show therapeutic efficiency on NSCLC cells to a certain degree. Unfortunately, the impact was suboptimal [28], thus making us curious about the potential of miR-133a as therapeutic strategy. Successive experiments are undergoing.

The molecular mechanisms between miR-133a and the tumorigenesis of NSCLC may be concerned with other targets. Moriya et al. [32] stated that miR-133a regulates ARPC5 and GSTP1 to perform a tumor-suppressive function. It remains a long way to go when it comes to the molecular mechanism of miR-133a and its target genes in NSCLC.

## Conclusions

The current research along with other related studies firmly suggest that miR-133a serves as a tumor-suppressive miRNA, which plays a crucial part in the oncogenesis and progression of human NSCLC. The downregulation of miR-133a indicates deterioration in NSCLC patients. MiR-133a might be quite a promising predictive biomarker as well as probable therapeutic strategy for NSCLC. Our team intends to undergo further *in vitro* and *in vivo* studies to illuminate the role and mechanism of miR-133a in the malignant phenotype of NSCLC cell lines.

## References

[1] Lee PN, Forey BA (2013). Indirectly estimated absolute lung cancer mortality rates by smoking status and histological type based on a systematic review. BMC Cancer..

[2] Hirose T, Murata Y, Oki Y, Sugiyama T, Kusumoto S, Ishida H (2012). Relationship of circulating tumor cells to the effectiveness of cytotoxic chemotherapy in patients with metastatic non-small-cell lung cancer. Oncol Res.

[3] Barrow TM, Michels KB (2014). Epigenetic epidemiology of cancer. Biochem Biophys Res Commun.

[4] De Mello RA, Araujo A, Hespanhol V, Reis RM (2013). Loci identified through genome-wide association studies and lung cancer risk: is there anything more?. Sao Paulo Med J.

[5] Gazala S, Pelletier JS, Storie D, Johnson JA, Kutsogiannis DJ, Bedard EL (2013). A systematic review and meta-analysis to assess patient-reported outcomes after lung cancer surgery. Scientific World Journal..

[6] Luo L, Dong LY, Yan QG, Cao SJ, Wen XT, Huang Y (2014). Research progress in applying proteomics technology to explore early diagnosis biomarkers of breast cancer, lung cancer and ovarian cancer. Asian Pac J Cancer Prev.

[7] Brothers JF, Hijazi K, Mascaux C, El-Zein RA, Spitz MR, Spira A (2013). Bridging the clinical gaps: genetic, epigenetic and transcriptomic biomarkers for the early detection of lung cancer in the post-National Lung Screening Trial era. BMC Med..

[8] Sen R, Ghosal S, Das S, Balti S, Chakrabarti J (2014). Competing endogenous RNA: the key to posttranscriptional regulation. Scientific World Journal..

[9] Callari M, Tiberio P, De Cecco L, Cavadini E, Dugo M, Ghimenti C (2013). Feasibility of circulating miRNA microarray analysis from archival plasma samples. Anal Biochem.

[10] Guz M, Rivero-Muller A, Okon E, Stenzel-Bembenek A, Polberg K, Słomka M (2014). MicroRNAs-role in lung cancer. Dis Markers..

[11] Daniels MG, Bowman RV, Yang IA, Govindan R, Fong KM (2013). An emerging place for lung cancer genomics in 2013. J Thorac Dis..

[12] Yasui W, Sentani K, Sakamoto N, Anami K, Naito Y, Oue N (2011). Molecular pathology of gastric cancer: research and practice. Pathol Res Pract.

[13] Chen Z, Xu L, Ye X, Shen S, Li Z, Niu X (2013). Polymorphisms of microRNA sequences or binding sites and lung cancer: a meta-analysis and systematic review. PLoS One.

[14] Oom AL, Humphries BA, Yang C (2014). MicroRNAs: novel players in cancer diagnosis and therapies. BioMed Res Int..

[15] Di Leva G, Briskin D, Croce CM (2012). MicroRNA in cancer: new hopes for antineoplastic chemotherapy. Ups J Med Sci.

[16] Hui A, How C, Ito E, Liu FF (2011). Micro-RNAs as diagnostic or prognostic markers in human epithelial malignancies. BMC Cancer..

[17] Fortunato O, Boeri M, Verri C, Moro M, Sozzi G (2014). Therapeutic use of microRNAs in lung cancer. BioMed Res Int..

[18] Del Vescovo V, Grasso M, Barbareschi M, Denti MA (2014). MicroRNAs as lung cancer biomarkers. World J Clin Oncol.

[19] Chiyomaru T, Enokida H, Tatarano S, Kawahara K, Uchida Y, Nishiyama K (2010). miR-145 and miR-133a function as tumour suppressors and directly regulate FSCN1 expression in bladder cancer. Br J Cancer.

[20] Kano M, Seki N, Kikkawa N, Fujimura L, Hoshino I, Akutsu Y (2010). miR-145, miR-133a and miR-133b: tumor-suppressive miRNAs target FSCN1 in esophageal squamous cell carcinoma. Int J Cancer.

[21] Zhao L, Wang H, Liu C, Liu Y, Wang X, Wang S (2010). Promotion of colorectal cancer growth and metastasis by the LIM and SH3 domain protein 1. Gut.

[22] Wu ZS, Wang CQ, Xiang R, Liu X, Ye S, Yang XQ (2012). Loss of miR-133a expression associated with poor survival of breast cancer and restoration of miR-133a expression inhibited breast cancer cell growth and invasion. BMC Cancer..

[23] Kawakami K, Enokida H, Chiyomaru T, Tatarano S, Yoshino H, Kagara I (2012). The functional significance of miR-1 and miR-133a in renal cell carcinoma. Eur J Cancer.

[24] Kojima S, Chiyomaru T, Kawakami K, Yoshino H, Enokida H, Nohata N (2012). Tumour suppressors miR-1 and miR-133a target the oncogenic function of purine nucleoside phosphorylase (PNP) in prostate cancer. Br J Cancer.

[25] Wang LK, Hsiao TH, Hong TM, Chen HY, Kao SH, Wang WL (2014). MicroRNA-133a suppresses multiple oncogenic membrane receptors and cell invasion in non-small cell lung carcinoma. PLoS One.

[26] Li Y, Li Y, Yang T, Wei S, Wang J, Wang M (2013). Clinical significance of EML4-ALK fusion gene and association with EGFR and KRAS gene mutations in 208 Chinese patients with non-small cell lung cancer. PLoS One.

[27] Butnor KJ, Beasley MB, Cagle PT, Grunberg SM, Kong FM, Marchevsky A (2009). Protocol for the examination of specimens from patients with primary non-small cell carcinoma, small cell carcinoma, or carcinoid tumor of the lung. Arch Pathol Lab Med.

[28] Chen G, Umelo IA, Lv S, Teugels E, Fostier K, Kronenberger P (2013). miR-146a inhibits cell growth, cell migration and induces apoptosis in non-small cell lung cancer cells. PLoS One.

[29] Chen G, Kronenberger P, Teugels E, Umelo IA, De Greve J (2012). Targeting the epidermal growth factor receptor in non-small cell lung cancer cells: the effect of combining RNA interference with tyrosine kinase inhibitors or cetuximab. BMC Med..

[30] Rong M, He R, Dang Y, Chen G (2014). Expression and clinicopathological significance of miR-146a in hepatocellular carcinoma tissues. Ups J Med Sci.

[31] Dang YW, Zeng J, He RQ, Rong MH, Luo DZ, Chen G (2014). Effects of miR-152 on cell growth inhibition, motility suppression and apoptosis induction in hepatocellular carcinoma cells. Asian Pac J Cancer Prev.

[32] Moriya Y, Nohata N, Kinoshita T, Mutallip M, Okamoto T, Yoshida S (2012). Tumor suppressive microRNA-133a regulates novel molecular networks in lung squamous cell carcinoma. J Hum Genet.

[33] Zheng DJ, Yu GH, Gao JF, Gu JD (2013). Concomitant EGFR inhibitors combined with radiation for treatment of non-small cell lung carcinoma. Asian Pac J Cancer Prev.

[34] Roy M, Luo YH, Ye M, Liu J (2013). Nonsmall cell lung cancer therapy: insight into multitargeted small-molecule growth factor receptor inhibitors. BioMed Res Int..

